# Supplementary material for: Prevention of post-traumatic stress disorder: Lessons learned from a terminated RCT of prolonged exposure
Source: PLoS One. 2021 May 24;16(5):e0251898. doi: 10.1371/journal.pone.0251898 (PMC8143412; doi:10.1371/journal.pone.0251898)
Supplement: S1 File — (DOCX) [file pone.0251898.s002.docx]

Early intervention after psychological trauma: Does it prevent Post-Traumatic Stress Disorder?

Principal investigator: Erik Andersson

Version: 1.0

**Abstract**

Traumatic events, such as traffic accidents, assaults and rapes, are unfortunately common phenomena in the population. Typical reactions for victims are flashbacks, increased anxiety and depressive symptoms. These symptoms usually disappear within a few months after the event, but for about 20%, these symptoms may develop into post-traumatic stress disorder (PTSD).

Although PTSD is relatively common, only a fraction of sufferers are detected by the health care system and detected cases are often left without proper treatment. PTSD is associated with increased risk of suicide and sick leave, and preventive work is clearly needed to lower the incidence of this disorder. Critically, as PTSD is the only mental health disorder where we have a known onset event, we should seize the opportunity to prevent the buildup of potentially long-standing symptoms.

Trauma Focused Cognitive-Behavior Therapy (TF-CBT) is the most promising intervention for preventing PTSD. TF-CBT is provided directly at the hospital emergency room, and has been shown in one trial to decrease the incidence of later PTSD. However, this recent and promising study acknowledged several limitations such as a small sample size, short follow-up time and lack of proper monitoring of treatment side effects.

In light of this, we propose a large-scale randomized trial (n=352), where patients attending an emergency room and have experienced a traumatic event are randomized to either TF-CBT or treatment-as-usual. We will measure PTSD symptoms, quality of life, treatment side effects, process data as well as health economic data up to 10 years.

If TF-CBT is found to be effective, it would mean that we can intervene on PTSD before it develops and we would have valuable knowledge about it´s cost-effectiveness and on the biological and psychological mechanisms that are at play. This would be of immense importance to the millions of people worldwide who will experience inevitable future accidents and traumatic events.

Introduction

**1 ABOUT POST-TRAUMATIC STRESS DISORDER**

1.1 PREVALENCE AND CLINICAL FEATURES

About 51-61% of the general population will experience a traumatic event at some point in their life (e.g. traffic accident, assault or rape).[^1^](#_ENREF_1) Typical reactions after a trauma are intrusive memories of the event, flashbacks, and depressive symptoms.[^2^](#_ENREF_2) These symptoms usually disappear within a few days or weeks after the event, but for about 20%, these symptoms develop into post-traumatic stress disorder (PTSD).[^3^](#_ENREF_3) PTSD is a mental disorder and means that the person who was exposed to trauma still re-experiences this traumatic event through intrusive memories at least one month after the event and these symptoms cause clinical significant distress.[^1^](#_ENREF_1)

PTSD is a detrimental condition for the sufferer and is associated with increased risk of suicide, drug- and alcohol dependence and sick leave.[^4^](#_ENREF_4) Additionally, PTSD-patients show increased somatic problems including both neurological-, vascular-, respiratory-, gastrointestinal and autoimmune diseases.[^5^](#_ENREF_5) PTSD is relatively common in Sweden with a life-time prevalence of 5.6%[^6^](#_ENREF_6) and recent numbers have shown that as much as 20-30% of the patients at the emergency trauma care at Karolinska Hospital, Stockholm, have clinical symptoms that warrants treatment.[^7^](#_ENREF_7) Thus, this is a serious societal problem.

1.2 TREATMENTS

The current psychological treatment for PTSD with best empirical support is Trauma Focused Cognitive-Behavior Therapy (TF-CBT). Briefly, in TF-CBT symptoms of PTSD are seen as signs of an incompletely processed memory, and that the memory is associated with high levels of fear and stress, which in turn drive avoidance behaviors that prevent the person from facing their memories and overcoming their problems. Thus, in TF-CBT the patient re-activates the memory repeatedly in a safe and non-stressful context (i.e., visualizes the event in their mind’s eye several times) for the fearful responses associated to the memory to gradually decline. As a consequence, the flashbacks and intrusions disappear. TF-CBT consists of 15 sessions that is focused on this process i.e. that the patient exposes him/herself to the fear memory and stay in that memory until the fear response is extinguished. TF-CBT for PTSD has been investigated in numerous trials with overall large effect sizes and remission rates averaging 55%-75%.[^8^](#_ENREF_8) Pharmacotherapy has also been tested in several trials but with substantially lower effect sizes compared with TF-CBT.[^9^](#_ENREF_9)

1.3 CURRENT BARRIERS TO TREATMENT

Despite the fact that PTSD is common, only a fraction of the persons suffering from this disorder are detected by the health care system. One example of this is the study by Al-Saffar where 115 psychiatric patients were interviewed and screened for PTSD. The authors found that about 20-30% of the psychiatric patients in this sample fulfilled the diagnostic criteria of PTSD, but none of them had actually received a PTSD-diagnosis from their physician.[^10^](#_ENREF_10) Additionally, although effective treatments for PTSD exist, many patients that are detected are still often left without proper treatment. In Sweden, less than 20% of clinicians use evidence based psychological treatment in their regular practice for PTSD.[^11^](#_ENREF_11) Thus, given the societal burden associated with PTSD and the problems with both detection and treatment, we clearly need to find alternative ways to lower the prevalence of this disorder.

1.4 THE POSSIBLE SOLUTION

The traditional psychiatric research paradigm has been to investigate the efficacy of a treatment package for patients who suffer from an already developed mental health disorder. Although this approach have been partly successful and helped a lot of patients, we have still not managed to substantially decrease the prevalence of any mental disorder.[^12^](#_ENREF_12)^,^[^13^](#_ENREF_13) An alternative approach here would instead be to develop preventive interventions i.e. to intervene on the disorder before it develops. Preventive care has been successful in physical medicine (e.g. cancer- or heart disease- research)[^12^](#_ENREF_12)^,^[^13^](#_ENREF_13) but has lagged considerably behind in the mental health field. As PTSD is the only mental health disorder where we have a known onset event, it is an obvious candidate for preventive interventions.

**2 PREVENTIVE INTERVENTIONS FOR PTSD**

2.1 PREVIOUS RESEARCH

There have been several attempts the last decade to pharmacologically intervene on PTSD before it develops. Four trials have found that early administration of hydrocortisone can make a small preventive effect but these trials also suffered form several methodological limitations. Other pharmacological have shown disappointing results.[^14^](#_ENREF_14)

For psychological treatments, only one intervention has so far proven to be effective in preventing the development of PTSD. This intervention is a shortened version of TF-CBT and is provided directly at the hospital emergency department about 12-24 hours after the trauma. This treatment is only 3 sessions long and is administered within a couple of weeks after experiencing trauma. The main point of giving TF-CBT acutely is to modify fear memories before they go through extensive consolidation during sleep cycle, thereby preventing the onset of PTSD.[^15^](#_ENREF_15) One recent randomized trial by Rothbaum et al. pilot tested this shortened version of TF-CBT on 137 trauma care patients (treatment was given at the emergency department) and found that this intervention indeed managed to decrease the incidence of later PTSD.[^16^](#_ENREF_16)

2.2 REMAINING RESEARCH QUESTIONS THAT NEED TO BE ANSWERED

Preliminary results from the Rothbaum et al. study are encouraging as it is the first study that has managed to show clinically meaningful effects in preventing PTSD. However, this study also experienced several limitations and there are still several research gaps that need to be filled. Below are the current research questions that are critical to answer in order to take the next step and implement TF-CBT in regular emergency care.

2.2.1 IS TF-CBT AN EFFICATIOUS AND SAFE TREATMENT IN PREVENTING PTSD?

One major limitation with the Rothbaum et al. trial was that it used a relatively small sample size (n=137) and this may question the reliability of the results. It is important to note that in these settings a sizeable portion of the participants will recover spontaneously and larger samples are needed as compared to treatments of chronic patients. Another limitation in their study was the short follow-up time of 12 weeks. This is problematic both from a scientific view as well as for policy makers because we do not know how this treatment stands in a 6- or 12-month period. Furthermore, the Rothbaum study also lacked systematic data collection on potential side effects that were associated with the treatment. This is an important issue as one psychological intervention for PTSD (i.e. “Debriefing”) has actually shown worsening of symptoms compared to treatment-as-usual.[^17^](#_ENREF_17) Thus, we need to do a large-scale trial with longer follow-up assessments and close monitoring of adverse events .

2.2.2 IS TF-CBT A COST-EFFECTIVE TREATMENT IN PREVENTING PTSD?

Another limitation with the Rothbaum et al. study was the lack of a health economical evaluation. From an implementation perspective, this is an important issue as policy makers need to know how much benefit an intervention do in relation to the costs associated with the intervention. Cost-effectiveness research has received increasing interest the last decades but lacks considerably behind in both psychiatric- as well as psychological science.[^18^](#_ENREF_18) Thus, one critical aspect here is to find out how much societal gains TF-CBT provides in relation to the economical costs.

2.2.3 HOW DO WE ACHIEVE SUCCESSFUL OUTCOME IN TF-CBT?

One final limitation with the Rothbaum et al. study was the lack of investigation of hypothesis driven underlying mechanisms. Revealing the mechanisms of psychological treatments are imperative in order to move the field forward.[^12^](#_ENREF_12)^,^[^13^](#_ENREF_13)^,^[^19^](#_ENREF_19) Thus, the key and also novel area that needs more research is about mediators of treatment response – that is the underlying mechanisms.

TF-CBT is based on “emotional processing theory” which stipulates that, in order to achieve complete emotional processing, the patient needs to a) active the fear memory (initial fear activation), b) stay in the fear memory until the fear subsides (within-session habituation) and c) repeat this procedure until the memory is no longer associated with fear (between-session habituation).[^20^](#_ENREF_20) Although previous studies have shown support for this pathway (i.e. initial fear activation 🡪 within-session habituation 🡪 between-session habituation 🡪 lower PTSD symptoms),[^20^](#_ENREF_20) it has never been tested within a preventive context.

Data on treatment mediators is important because it would provide us with knowledge of how the treatment achieves its effects. For instance, if patients who do not succeed with initial fear activation 🡪 within-session habituation 🡪 between-session habituation would have worse treatment response, it would mean that we could increase the amount of support for these patients and maximize the treatment dose. In addition, research is also lacking on other emotions such as disgust, not-just-right emotions or shame/anger.[^21^](#_ENREF_21) Research about this would not only give us better treatment effects but, even more importantly, it would give us new knowledge about psychological reactions and how to manipulate memory consolidation in the immediate aftermath after experiencing trauma.

**3 THE NEXT RESEARCH STEP**

Our research group has completed over 20 randomized controlled trials (both psychological and pharmacological treatments) for different mental health disorders. These trials have all followed a classical psychiatric outline i.e. investigate the efficacy of a treatment package for patients who suffer from an already developed mental health disorder. However, the next logical step is now to 1) create preventive treatments, 2) understand how these treatments work in terms of mechanisms and 3) gain knowledge for whom does it work for. This focus on process research and exploring the psychological and biological mechanisms has also recently been pointed out by the US national institute of mental health (NIMH) in the new “research domain criteria” (RDoC).[^12^](#_ENREF_12)^,^[^13^](#_ENREF_13)

3.1 THE RESEARCH TEAM

In order to fill the previously mentioned research gaps, we have created a research group with experts in trauma, treatment of PTSD and process research:

**Emily Holmes** is a clinical psychologist and professor at the University of Oxford and currently Guest Professor at Karolinska Institutet. Professor Holmes has extensive experience in treatment studies of PTSD and also mechanisms research. She is regarded as on of the top experts in PTSD and bipolar disorder. She also has experience of running research in the emergency department, of basic emotion research and also of clinical supervision of trauma therapy – all which will be contributed to the current project.

**Filip Arnberg** is a clinical psychologist, post doc at Stockholm Stress Research Institute and also assistant director of the National Centre for Disaster Psychiatry at Uppsala University. Dr. Arnberg has an extensive track record in epidemiological research studies on PTSD and clinical work with these patients, and will provide this project with important competence on the research design and procedures.

**Maria Bragesjö** (planned PhD student in this project) is the only clinician Europe who is a certified trainer and supervisor in trauma focused cognitive behavior therapy (TF-CBT) for post-traumatic stress disorder (PTSD). Bragesjö is regarded as one of the top experts in the world in treating PTSD with TF-CBT and has been headhunted to Karolinska Sjukhuset with the specific aim is to start a unit that delivers psychological interventions in trauma care (this project).

3.2 OUR GENERAL AIM

We see a need for research on TF-CBT as a preventive treatment along as well as to extend this well-defined treatment paradigm to investigate mediators of treatment response and biomarkers, with additional analyses on health economical aspects. As pointed out by the chief director at NIMH in a recent paper in JAMA Psychiatry,[^12^](#_ENREF_12) focus on mediators is especially important as it allows a trial to be informative even if the intervention would not show any incremental effects i.e. we can investigate if we were able to affect the mediator and if the mediator in turn affected the outcome.

**4 PURPOSE OF THE STUDY**

Prevention of stress symptoms after a traumatic event is a major goal for psychological treatment innovation and science alike. The main purpose of this study is to test if TF-CBT provided soon after trauma at a Hospital emergency department can be effective in decreasing the incidence of PTSD. We also want to investigate the active psychological and biological mechanisms that are underlying this intervention.

4.1 RESEARCH QUESTIONS

1. Is TF-CBT an effective treatment in preventing the development of PTSD symptoms?
2. Is TF-CBT a cost-effective treatment in preventing the development of PTSD symptoms?
3. What are the mediators of treatment response; i.e. the underlying psychological and biological mechanisms?

Methods

**1. IS TF-CBT AN EFFECTIVE TREATMENT IN PREVENTING THE DEVELOPMENT OF PTSD SYMPTOMS?**

1.1 OVERALL DESIGN AND PROCEDURE

The design is a randomized controlled trial, where patients in the emergency department who have experienced a traumatic event according to the A-criteria in the DSM-5 (e.g. car crash, rape), are randomized to receive either TF-CBT or to placebo. The placebo group will receive basic written information about PTSD and how treat these symptoms. Thus, the rationale for the placebo arm is to provide the patient with a sham treatment to rule out any major placebo effects. Given the estimated effect sizes in the previous trial by Rothbaum et al. we would need 352 patients (176 in each group) in order to detect a standardized effect size (Cohen´s *d*) of 0.3 (*p* = .05, 80% power). We also want to include additional 10 participants in the beginning of the trial just to test the study procedures.

Exclusion criteria are the following: a) Do not want to participate in the study, b) Ongoing intoxication (e.g. due to alcohol), c) Low cognitive capacity, d) Other serious psychiatric comorbidity (ongoing psychotic symptoms or symptoms from bipolar 1, suicidal ideation)

We will screen all trauma patients at Traumacentrum Karolinska (about 1500 patients per year). Psychiatric interviews will be conducted by a licensed psychologist at the emergency department or any related clinic (e.g. if the patient has been transferred to another clinic). Patients who fulfill the A-criterion in DSM-5 (i.e. they have experienced a highly stressful event threatening physical or psychological integrity) will be asked to participate in the study. Informed consent will be delivered both as written text as well as explained by the study personnel. Baseline assessments (shown below) will then be conducted.

After the patients have signed the informed consent and completed the baseline assessments, they are randomized to either TF-CBT or placebo. Block randomization will be done beforehand by an independent party (Karolinska Trial Alliance; KTA) on an 1:1 ratio.

Participants randomized to TF-CBT will receive a brief 3-session intervention provided within 72 hours after the accident. In this treatment, the patient is instructed to systematically expose him/herself to the fearful memory of the event and stay in this memory until the anxiety has attenuated. By doing this exposure repeated times, the association between the memory and fear response is hypothesized to be gradually extinguished. The placebo group will receive sham treatment i.e. written information about PTSD and how to treat it but without any therapist-guided interventions.

We will measure PTSD symptoms using the Clinician Administered PTSD Scale (CAPS; the gold standard for PTSD assessment) and Posttraumatic Diagnostic Scale,[^22^](#_ENREF_22)^,^[^23^](#_ENREF_23) depression using the Montgomery Åsberg Depression Rating Scale – Self rated (MADRS-S),[^24^](#_ENREF_24) quality of life using the EQ-5D[^25^](#_ENREF_25) and treatment side effects using the Safety Monitoring Uniform Report Form (SMURF)[^26^](#_ENREF_26) at baseline (i.e. before treatment allocation), 2-, 6-, 12-, 24-, 48- and 120 months using blinded assessors. Primary endpoint is set to 6-months after the trauma. Patients unable to come to the clinic for follow-up assessments will be interviewed via telephone or skype.

The primary outcome (significant PTSD symptoms assessed by the CAPS) will be analyzed using risk ratios and numbers-needed-to-treat with 95% confidence intervals. Continuous data will be analyzed in a mixed-effects model adjusting for data attrition (covariance and residual structure will be set based on the best model fit of the data) using intention-to-treat design. Effect sizes for continuous measures will be estimated using Cohen´s *d*. Assessor´s blindness will be also tested (each assessor will guess treatment allocation for each participant and this will be tested in a logistic regression with guess as independent variable and allocation as dependent variable). A flowchart of the treatment study is shown in the attached figure.

1.2 DETAILED METHODOLOGCAL QUALITY PROCEDURES

1.2.1 BLINDING AND CODE BREAKING

We will use sealed envelopes which will be kept safe and accessible only to persons authorized to unblind. A decoding list containing the treatment codes will be kept at the clinic. Randomization allocation for each participant will be made available only after signed consent. Block size of each randomization will be blinded to investigators by KTA. The circumstances under which the code may be broken to other than the treating psychologist are life-threatening emergencies. Any broken code will be logged with name, signature, date, the number of the participant concerned and reason for breaking the code on the code envelope.

1.2.2 CRITERIA FOR WITHDRAWAL

*1.2.2.1 Criteria for mandatory discontinuation of treatment*

- Consent withdrawal by the patient
- High suicide risk according to investigator’s judgment or any suicide attempts during the study whatever its severity
- Worsening of PTSD symptoms, which in the opinion of the investigator requires an adaptation of treatment not compatible with the protocol or requires hospitalization.

*1.2.2.2 Other possible reasons for premature discontinuation of treatment*

- Any adverse event or circumstances justifying the discontinuation of the treatment in the investigator’s opinion.
- Treatment failure
- Protocol deviation which jeopardizes the patient’s safety

We will record the reason and the exact time of the premature discontinuation of treatment in the case report form (CRF). If more than one reason is given, we will indicate the main reason according to good clinical practice (GCP).

1.2.3 SIDE EFFECTS PROCEDURES

An Adverse Event (AE) in this trial is any untoward medical occurrence regardless of its causal relationship with TF-CBT. AE is assessed at each clinician visit using the SMURF and is classified by the investigator as mild, moderate or severe:

- **Mild:** Acceptable. The subject is awareness of symptoms or signs, but they are easy tolerated.
- **Moderate:** Disturbing. The AE is discomfort enough to interfere with usual daily activity.
- **Severe:** Unacceptable. The subject is unable to work or to do usual daily activities (e.g. death, life-threatening, significant incapacity).

If the AE is regarded as severe, it will be defined as a Serious Adverse Event (SAE). SAEs will be reported in detail to Socialstyrelsen (responsible for side effects in psychological treatments) and will also be stored in the CRF.

Additionally, each AE is to be classified with Assessment of Causality (stored in the CRF):

- **Unlikely:** The event is most likely related to an aetiology other than TF-CBT.
- **Possible:** A causal relationship is conceivable and cannot be dismissed.
- **Probably:** Good reason and sufficient documentation to assume a causal relationship.

If an SAE is at least possibly related to TF-CBT treatment, then it is also to be classified by the investigator as expected or unexpected. An unexpected SAE is defined as a Suspected Unexpected Serious Adverse Reaction (SUSAR). It is expected if it is already known from earlier CBT trials or is mentioned in relevant documents. This will also be directly reported to Socialstyrelsen with follow-up information describing the outcome of the SAE and action taken will be reported as soon as it is available.

In addition, three interim analyses will be conducted by KTA to ensure that the TF-CBT group is not associated with any significant worsening of symptoms. Thus, we have taken extensive steps in order to monitor and record any possible side effects associated with TF-CBT.

**2 IS TF-CBT A COST-EFFECTIVE TREATMENT IN PREVENTING THE DEVELOPMENT OF PTSD SYMPTOMS?**

2.1 OVERALL DESIGN

Patients from the randomized trial (research question 1) will be followed in the patient register (Patientregistret), the prescription register (Läkemedelsregistret) and the longitudinal integrated data-base for health insurance and work-related research (LISA - Longitudinell integrationsdatabas för sjukförsäkrings-och arbetsmarknadsstudier) Costs will be analyzed using a societal perspective i.e. including both sick leave, hospitalizations, medical use etc. and analyzed in relation to outcome (i.e. PTSD symptoms and also quality-adjusted life years which are assessed in the randomized trial using the CAPS and EQ-5D).

2.2 COST ESTIMATIONS

We will use national tariffs to estimate costs form different forms of health care visits as we have done previously in other cost-effectiveness studies (see publication list for more details). Productivity losses will be estimated using gross earning data from each patient. The treatment costs (i.e. the cost of having a full time psychologist at the emergency department) will be included in the cost estimation.

2.3 COST-EFFECTIVENESS ANALYSES

Cost-effectiveness comparisons will be analyzed using incremental cost-effectiveness ratios as well as the net benefit approach with different willingness-to-pay scenarios. Cost-effectiveness ratios are calculated as following: (Δ^C1^ – Δ^C2^)/(Δ^E1^ – Δ^E2^), where Δ^C1^ – Δ^C2^ is the difference in cost change (from pre- to follow-up) between the two treatment groups. Δ^E1^ – Δ^E2^ refers to the corresponding difference in clinical outcome (i.e. PTSD vs. no PTSD). Because cost-effectiveness is a relative term i.e. an intervention is only cost-effective depending on how much the society is willing to pay for one unit of improvement, we will also use the ”net benefit approach”, which estimates the cost-effectiveness depending on different societal willingness-to-pay values for one unit of improvement. The net benefit of each individual in this trial will be calculated according to the following formula: (λ x E) – Δ^C^ where λ is the willingness to pay (i.e. the different values that the society is willing to pay for one unit of improvement), E is the efficacy (i.e. PTSD status or gain on the EQ-5D), and Δ^C^ is the cost change from baseline to primary endpoint (6-months). We will calculate individual net benefits by assuming different values for λ and a regression model with non-parametric bootstrapping (one thousand replications) to estimate be difference between TF-CBT treatment-as-usual. Cost-effectiveness acceptability curves will be used to get a visual estimate of the total net benefit of TF-CBT.

**3 WHAT ARE THE MEDIATORS OF TREATMENT RESPONSE; I.E. THE UNDERLYING PSYCHOLOGICAL AND BIOLOGICAL MECHANISMS?**

3.1 PROCEDURES AND EQUIPMENT

In order to test emotional processing theory in a preventive treatment context, we will use process data from the randomized trial (research question 1). In each exposure session, the psychologist will ask the patient to give a detailed description of the traumatic event, thereby activating the fear memory. Activation of fear memory elicits an associated fear reaction which we will measure with both the subjective units of distress scale,[^27^](#_ENREF_27) heart rate (measured through standard equipment at the hospital) and skin conductance. The patient is then instructed to hold the fear memory in their mind’s eye and describe it in detail for the psychologist until the fear response associated with the memory has attenuated (also known as “within session habituation”).

We will measure time to habituation and also the individual trajectories for each session i.e. we will assess whether the anxiety habituate in an u-shaped curve as suggested by some research[^28^](#_ENREF_28) or if it has a waxing and waning trajectory as suggested by other studies.[^29^](#_ENREF_29) Process data will be collected each session (in total 3 sessions).

3.2 STATISTICAL ANALYSES

We will then conduct a path analysis and test whether outcome (i.e. PTSD symptoms) is dependent on the following causal steps: initial fear activation 🡪 within session habituation 🡪 between session habituation. As some research has suggested that type of emotional response matter, we will also collect additional data on the primary emotional response (disgust, anger, and not-just-right emotions) on disgust, anger and not-just-right emotions factor variables in a predictor model.

# Ethical considerations

Patients will receive both written and verbal information about the study. Participation is voluntarily. It will be possible to participate in the treatment trial but not in the genetic investigation. The collaborator (Holmes) has research experience of informed consent in an Emergency Department, and procedures for working with the medical team more broadly.

Source data will be quality monitored by an independent party (KTA) according to the Helsinki Declaration. We will set the quality control as equal to a pharmacological trial.

We will also conduct at least three interim analyses during treatment. The reason for this is to monitor potential side effects associated with treatment such as worsening of symptoms. The interim analyses will be conducted by KTA and handled according to good clinical practice.[^30^](#_ENREF_30) If TF-CBT would be shown to cause any serious side effects, we will make an assessment together with external experts if the study should needs to be stopped beforehand. This is an important issue from an ethical perspective as one psychological intervention for PTSD (i.e. “Debriefing”) has actually shown worsening of symptoms compared to treatment-as-usual.[^17^](#_ENREF_17) However, given the positive Rothbaum trial this is unlikely for this new intervention.

# Working plan

Preparation for the study

Maria Bragesjö (planned PhD student of this project) has the last quarter established routines at the emergency department and shown that it is feasible to implement TF-CBT within this context. Maria is the only certified TF-CBT trainer in Europe. Holmes (collaborator) has just completed an RCT in an emergency department of a different intervention (a computer game) demonstrating the feasibility of recruiting patient soon after trauma, working within a busy hospital setting and recruitment rates needed for the successful completion of this trial in the time frame. We have also extensive collaboration with leading figures in the trauma clinic at Karolinska University Hospital (see below). The study is sanctioned also by Psykologkliniken who will be the main sponsor of this study and enable infra structure, personnel etc. We have also translated and validated the PTSD outcome questionnaires and interviews for a Swedish population. Thus, preliminary results from Rothbaum et al. suggest that TF-CBT can prevent PTSD and our research group has the resources and competence to investigate this in more detail.

Time plan

- 2015 Consolidation of the treatment manual and establishing routines at Traumacentrum Karolinska
- 2016-2017 Recruitment, treatment with TF-CBT
- 2017-2019 Follow-up assessments. Primary endpoint completed.
- 2019- Write papers
- 2020- Publish papers
- 2020🡪 Long term follow-up assessments (not within the scope of this application)

# Scientific implications

This project holds important scientific implications:

First, there is to date only one early stage randomized controlled trial in the USA showing that PTSD can be prevented through TF-CBT. This current study is unique in providing an adequately powered test of the hypothesis using a larger sample size, allowing longer-term follow-up assessments, as well as appropriate monitoring of treatment side effects. In addition, the health economical analyses will reveal how much the society needs to pay for one non-case of PTSD. This study is therefore set to be the definitive trail internationally.

Second, a unique aspect of this clinical research study is that we will examine the underlying mechanisms of the treatment, drawing on work in basic neuroscience and cognitive theory. The intervention is hypothesized to modify the trauma memory, and thus to prevent the development of PTSD symptoms, before any major memory consolidation has taken place (i.e. sleep on night one[^31^](#_ENREF_31)) which would otherwise cement the traumatic memories in a maladaptive way. It would therefore be of great scientific value to see if the stipulated effect of the emotional processing theory, which TF-CBT draws upon, applies within this preventive context in a non-psychiatric sample. We will also investigate the role of different emotions and their consequences in treatment outcome. There is a lot of research on fear extinction but, as many other emotions are involved in PTSD as well as other mental disorders, the next step would also to investigate the role of disgust, shame etc. and see if these emotions follow the same path as fear extinction. Research about emotional memory consolidation and treatment mediators would give us highly valuable information both about the etiology of PTSD, and also information on how to treat PTSD in the most effective way with psychological interventions. If we know how our treatment works optimally, we can implement additional instruments and procedures that ensure that we activate the working mechanisms in this treatment. Critically, this study will also be able to demonstrate whether treatment success (or failure) is contingent on the hypothesized mechanisms. Therefore this study will be among the first to link the basic laboratory work on memory consolidation to an applied real world setting.

These additional aspects of the study are useful step in tailoring preventative treatments, will also add value to the study, regardless of whether the treatment if successful or not (which in itself is a critical question to test) contributing both the basic and clinical science base.

# Societal implications

In the last decade, both the Swedish Government and the National Board of Health have underscored the importance of using a preventive health care approach.[^32^](#_ENREF_32)^,^[^33^](#_ENREF_33) PTSD is a major public health concern with long-term sequelae, and given the signiﬁcant public health impact of PTSD, there is a need for research on brief and cost-effective interventions that are easy to disseminate.

If TF-CBT would be shown effective, it would mean that the health care system could access and implement a new and brief intervention to the treatment armament and potentially “vaccinate” many trauma patients from developing PTSD. The cost-effectiveness aspect of the study will also provide us with important data whether TF-CBT also can decrease the burden on the social welfare system as well as psychiatry and addiction care. Thus, this project also holds important precision prevention features, similar to what has been done in cancer-, heart disease- or diabetes- research,[^12^](#_ENREF_12)^,^[^13^](#_ENREF_13) moving this for the first time in Sweden to psychological treatment research and mental health. Lastly, the data from the mediation analysis would provide us with better knowledge what to focus on in psychological treatment, hence potentially improve these interventions and thereby minimize treatment failures for many patients.

The prospect of being able to prevent PTSD by means of a psychological intervention poses great challenges, but the rewards are of immense importance: not only to the thousands of people in Sweden who every year are victims of road traffic accidents or interpersonal violence, but also to the millions of people worldwide who will experience inevitable future traumatic events.

Collaborations:

**Kerry Ressler**, associate professor at Emory University Atlanta USA, is an external consultant in the current project. Professor Ressler is one of the top leading researchers on translational models of fear extinction, and was also one of the main authors behind the previous RCT on TF-CBT conducted in Atlanta, USA.[^16^](#_ENREF_16) We have previously worked with Ressler in a randomized pharmacotrial with great success. More info at <http://resslerlab.com>. Ressler will serve the function as an external consultant in the current project.

**Carl Magnus Wahlgren,** associate professor at KI, surgeon and head of research at Trauma Centrum Karolinska. Dr. Wahlgren will enable infrastructure at the emergency department for this project.

Two other important figures in enabling infrastructure is our collaborators **Peter Sackey** (associate professor and surgeon on intensive trauma care at Karolinska Universitetssjukhuset) and **Olof Brattström**, PhD and specialist in anaesthesia.

**Brjánn Ljótsson** is a clinical psychologist and associate professor at KI. Dr. Ljótsson has extensive experience of conducting randomized trials and his main focus has been on process research and mediation analyses. Dr. Ljótsson will provide his expertise on the process studies in this project.

**Erik Hedman,** clinical psychologist and associate professor at KI, has conducted numerous clinical trials. Dr. Hedman has extensive experience in running clinical trials and then implement them in regular health care. He is responsible for the implementation of psychological treatments in Sweden´s largest primary care clinic. Dr. Hedman will provide this project with expertise in both running clinical trials and also the implementation process in regular care.

**References**

**1.** Kessler RC. Posttraumatic stress disorder: the burden to the individual and to society. *The Journal of clinical psychiatry.* 2000;61 Suppl 5:4-12; discussion 3-4.

**2.** Darves-Bornoz JM, et al. Main traumatic events in Europe: PTSD in the European study of the epidemiology of mental disorders survey. *Journal of traumatic stress.* 2008;21:455-62.

**3.** Barth J, et al. Posttraumatic stress disorders and extent of psychosocial impairments five years after a traffic accident. *Psycho-social medicine.* 2005;2:Doc09.

**4.** Kessler RC, et al. Posttraumatic stress disorder in the National Comorbidity Survey. *Archives of general psychiatry.* 1995;52:1048-60.

**5.** McFarlane AC, et al. Physical symptoms in post-traumatic stress disorder. *Journal of psychosomatic research.* 1994;38:715-26.

**6.** Frans O, et al. Trauma exposure and post-traumatic stress disorder in the general population. *Acta psychiatrica Scandinavica.* 2005;111:291-9.

**7.** Brattström O, et al. Psychiatric symptoms after surgical trauma: A cohort study. In prep.

**8.** van Minnen A, et al. Prolonged exposure in patients with chronic PTSD: predictors of treatment outcome and dropout. *Behaviour research and therapy.* 2002;40:439-57.

**9.** Hoskins M, et al. Pharmacotherapy for post-traumatic stress disorder: systematic review and meta-analysis. *The British journal of psychiatry : the journal of mental science.* 2015;206:93-100.

**10.** Al-Saffar S, et al. Long-term consequences of unrecognised PTSD in general outpatient psychiatry. *Social psychiatry and psychiatric epidemiology.* 2002;37:580-5.

**11.** Deacon BJ, et al. Therapist barriers to the dissemination of exposure therapy. In: Storch EA MD, ed. *Handbook of treating variants and complications in anxiety disorders*: Springer; 2013:363-73.

**12.** Insel TR, et al. National institute of mental health clinical trials: New opportunities, new expectations. *JAMA psychiatry.* 2014;71:745-6.

**13.** Cuthbert BN, et al. Toward the future of psychiatric diagnosis: the seven pillars of RDoC. *BMC medicine.* 2013;11:126.

**14.** Amos T, et al. Pharmacological interventions for preventing post-traumatic stress disorder (PTSD). *Cochrane Database Syst Rev.* 2014;7:CD006239.

**15.** Cain CK, et al. Targeting memory processes with drugs to prevent or cure PTSD. *Expert opinion on investigational drugs.* 2012;21:1323-50.

**16.** Rothbaum BO, et al. Early intervention may prevent the development of posttraumatic stress disorder: a randomized pilot civilian study with modified prolonged exposure. *Biological psychiatry.* 2012;72:957-63.

**17.** Rose S, et al. A systematic review of single-session psychological interventions ('debriefing') following trauma. *Psychotherapy and psychosomatics.* 2003;72:176-84.

**18.** Drummond MF. *Methods for the economic evaluation of health care programmes.* 3rd ed. ed. Oxford: Oxford University Press; 2005.

**19.** Holmes EA, et al. Psychological treatments: A call for mental-health science. *Nature.* 2014;511:287-9.

**20.** Foa EB, et al. The Impact of Fear Activation and Anger on the Efficacy of Exposure Treatment for Posttraumatic-Stress-Disorder. *Behavior Therapy.* 1995;26:487-99.

**21.** OLATUNJI BO, et al. *Disgust and psychiatric illness: have we remembered?* Vol 1902007.

**22.** Foa EB, et al. The validation of a self-report measure of posttraumatic stress disorder: The Posttraumatic Diagnostic Scale. *Psychological assessment.* 1997;9:445-51.

**23.** Weathers FW, et al. *The Clinician-Administered PTSD Scale for DSM-5 (CAPS-5).* National Center for PTSD 2013.

**24.** Svanborg P, et al. A new self-rating scale for depression and anxiety states based on the Comprehensive Psychopathological Rating Scale. *Acta psychiatrica Scandinavica.* 1994;89:21-8.

**25.** Rabin R, et al. EQ-SD: a measure of health status from the EuroQol Group. *Ann. Med.* 2001;33:337-43.

**26.** Bostic J. EVALUATION OF MEDICATION SIDE EFFECTS (derived from the Safety Monitoring Uniform Report Form [SMURF]). In: Hospital MG, ed2007.

**27.** Wolpe J. *The Practice of Behavior Therapy.* New York: Pergamon Press,; 1969.

**28.** Foa EB, et al. The impact of fear activation and anger on the efficacy of exposure treatment for posttraumatic stress disorder. *Behavior Therapy.* 1995;26:487-99.

**29.** Rowe MK, et al. Effects of varied-stimulus exposure training on fear reduction and return of fear. *Behaviour research and therapy.* 1998;36:719-34.

**30.** Pocock SJ. Interim analyses for randomized clinical trials: the group sequential approach. *Biometrics.* 1982;38:153-62.

**31.** Porcheret K, et al. Psychological impact of an analogue traumatic event reduced by sleep deprivation. *Sleep.* in press.

**32.** Socialstyrelsen. Så kan sjukvården förebygga sjukdom. 2012.

**33.** Regeringen. *En förnyad folkhälsopolitik.* Socialdepartmentet;2008.
