# Supplementary material for: Prevention of post-traumatic stress disorder: Lessons learned from a terminated RCT of prolonged exposure
Source: PLoS One. 2021 May 24;16(5):e0251898. doi: 10.1371/journal.pone.0251898 (PMC8143412; doi:10.1371/journal.pone.0251898)
Supplement: Attachment — Submitted filename: renamed_0eebc.docx [file pone.0251898.s005.docx]

**COMMENTS – Manuscript PONE-D-20-01989**

Title: “Prolonged Exposure as early intervention in an emergency department context: lessons learned from a terminated RCT”

**Important note: This review pertains only to ‘statistical aspects’ of the study and so** **‘****clinical aspects’ [like medical importance, relevance of the study,** **‘clinical significance and implication(s)’ of the whole study, etc.] are to be evaluated [should be assessed] separately/independently.**

Since this paper highlight obstacles and lessons learned from a terminated RCT / feasibility work relevant for preventive psychological interventions for PTSD in emergency settings, the title should indicate it (inclusion of word PTSD is must), in my opinion. Present title gives the impression that the paper is about methodology ‘regarding how to learn lessons from any terminated RCT’ in general. Look at the ‘objectives’ given in line 25-28.

This RTC is terminated even before recruiting 10% of targeted (minimum required) sample size of 352 participants [32/352=9.09% or 22/352=6.25%], so that the lessons learned from this terminated RCT data are not much meaningful. As said in lines 35-37 “One important finding was the high degree of attrition: only 78% and 34% respectively came back for the two months and six-months assessments” does that mean that although recruitment stopped, data collection continued?

Is it correct to combine data from the pilot study and whatever available from terminated RCT? as said in lines 81-83 [The sample reported throughout this paper is the pooled sample of 10 participants from the pilot study and the first 22 from the RCT]. Please check typing error in sentence in lines 117-8 that “Inter-rater agreement was excellent (Inter Class Correlation between 0.91-0.98).” which should be “Inter-rater agreement was excellent (Intra-Class Correlation Coefficient between 0.91-0.98).” because we assess Inter-rater agreement by Intra-Class Correlation Coefficient (and not by Inter Class Correlation). Further, note that there is only one value of Intra-Class Correlation Coefficient and a range as given. If you intend to mean differently, please clarify.

I believe that ‘Procedures’ (given in lines 96-111) & subsequent account {like Measures, etc.} are part of ‘protocol’ [because the trial is terminated], in that case ‘The study started recruitment 18^th^ of April 2017 and was terminated 11^th^ November 2017’ (lines 110-11) means it took more than six months to recruit 22 subjects {or was that also planned?}. Check the ‘tense’ of the entire text.

In my considered opinion [despite the fact which is highly appreciable that tools used (or proposed to be used) are very appropriate] whatever authors want to communicate [definitely important to communicate], could be communicated in just a letter-to-editor or brief communication (and not a full-length paper/article).
