# Supplementary material for: Prevention of post-traumatic stress disorder: Lessons learned from a terminated RCT of prolonged exposure
Source: PLoS One. 2021 May 24;16(5):e0251898. doi: 10.1371/journal.pone.0251898 (PMC8143412; doi:10.1371/journal.pone.0251898)
Supplement: Attachment — Submitted filename: Response to reviewers .docx [file pone.0251898.s006.docx]

PONE-D-20-01989
Dear Reviewer #1 and Reviewer #2,

First, we want to thank you for valuable comments. We have revised the manuscript accordingly and we think that it is greatly improved. Below follows a detailed description of the changes made point by point:

**Reviewer #1**

1. Since this paper highlight obstacles and lessons learned from a terminated RCT / feasibility work relevant for preventive psychological interventions for PTSD in emergency settings, the title should indicate it (inclusion of word PTSD is must), in my opinion. Present title gives the impression that the paper is about methodology ‘regarding how to learn lessons from any terminated RCT’ in general. Look at the ‘objectives’ given in line 25-28.

*Response: We thank Reviewer #1 for this thoughtful comment and we have incorporated the word PTSD in the title of the manuscript (page 1, Prevention of Post-Traumatic Stress Disorder: Lessons Learned from a Terminated RCT of Prolonged Exposure).*

2. This RTC is terminated even before recruiting 10% of targeted (minimum required) sample size of 352 participants [32/352=9.09% or 22/352=6.25%], so that the lessons learned from this terminated RCT data are not much meaningful.

*Response: We agree that the outcome data come with great uncertainty and we have elaborated on this further in the limitation section (page 18, first paragraph). As highlighted by Reviewer #1 in point 6 below, it took us nearly six months to recruit 22 participants. The study started out with a fair recruitment rate, 30% of the daily intake of trauma patients at the ED were included, but this figure declined swiftly to only 2.8% of trauma patients. This significant decline was due to major organizational changes at the recruiting site and was not expected. Although the number of included participants compared to the target sample was relatively low, we argue that the long recruitment period and sudden shift in recruitment rate provides the reader with important data on the role of organizational factors in early provided psychological interventions after trauma. Also, we do not judge 22-32 subjects as a meaningless sample size but sufficient to say something about the feasibility of using prolonged exposure as an early intervention in a hospital context. We agree with Reviewer #1 that statistical analysis of the sample may not be meaningful, and hence we have not conducted any such analyses. However, in line with Reviewer #2 below, we think the study still holds important lessons for both clinicians and researchers working with individuals early after exposure to trauma.*

3. As said in lines 35-37 “One important finding was the high degree of attrition: only 78% and 34% respectively came back for the two months and six-months assessments” does that mean that although recruitment stopped, data collection continued?

*Response: Yes, this is correct, as recruitment stopped, we continued the follow up as stated in the verbal and signed informed consent with the included participants. We deemed this to be best ethical practice as participants expected to receive a clinician visit (with the possibility to be offered further treatment, if there would be any need, for mental health issues). We agree with Reviewer #1 that this needs further clarification and we have added information about this on page 5, last paragraph, continuing on page 6.*

4. Is it correct to combine data from the pilot study and whatever available from terminated RCT? as said in lines 81-83 [The sample reported throughout this paper is the pooled sample of 10 participants from the pilot study and the first 22 from the RCT].

*Response: Because the trial was terminated early, we decided to include also the feasibility data from the pilot study. The participants in the pilot study underwent clinical procedures and routines that were identical to the participants in the RCT. Including participants also from this part of the larger study gave us greater precision to investigate feasibility aspects of using prolonged exposure as an early intervention in a hospital context. We think this improves the quality of the manuscript significantly but we are open to revise this if the Editor thinks differently. We have added a discussion of this under Limitations (page 18, first paragraph*).

5. Please check typing error in sentence in lines 117-8 that “Inter-rater agreement was excellent (Inter Class Correlation between 0.91-0.98).” which should be “Inter-rater agreement was excellent (Intra-Class Correlation Coefficient between 0.91-0.98).” because we assess Inter-rater agreement by Intra-Class Correlation Coefficient (and not by Inter Class Correlation). Further, note that there is only one value of Intra-Class Correlation Coefficient and a range as given. If you intend to mean differently, please clarify.

*Response: We thank reviewer #1 for pointing us this typing error in the manuscript. The inter-rater agreement is assessed by the Intra-Class Correlation Coefficient. We have changed this in the manuscript (page 6, last sentence). We have also clarified that the range given is from multiple assessment sessions.*

6. I believe that ‘Procedures’ (given in lines 96-111) & subsequent account {like Measures, etc.} are part of ‘protocol’ [because the trial is terminated], in that case ‘The study started recruitment 18th of April 2017 and was terminated 11th November 2017’ (lines 110-11) means it took more than six months to recruit 22 subjects {or was that also planned?}.

*Response: We have structured the manuscript closely to standard reporting of RCT:s in order to facilitate the reading of the manuscript. We therefore include standard headings in the Methods section, although we do see that it might make sense to revise the structure. Nevertheless, we would suggest to keep the current structure. As to the question from the Reviewer on the prolonged recruitment, we have clarified in the study progression paragraph in the Results section that, “The recruitment continued for six months as there was uncertainty as to whether the decline in eligible patients would become permanent, however, the low inclusion rate due to the organizational changes ultimately led to the discontinuation of the study “(page 11, first paragraph). Also note, as we point to in the manuscript, that the trial highlights the difficulty in recruiting participants in this early stage after exposure to trauma. Problems in recruiting have occurred also in other similar trials testing early intervention for patients who experienced trauma, with a low proportion of eligible participants among patients [1-3]. For example, the more recent trial by the Rothbaum et al research group investigating early provided prolonged exposure was terminated before reaching the target number of participants, leaving the study underpowered [2]. We suggest that an important lesson is that this is a factor to consider overall when planning early intervention studies and clinical routines.*

7. Check the ‘tense’ of the entire text.

*Response: We have re-read the manuscript with this in mind and adjusted the tense accordingly, and made additional language edits – we hope that the language now is up to standard.*

8. In my considered opinion [despite the fact which is highly appreciable that tools used (or proposed to be used) are very appropriate] whatever authors want to communicate [definitely important to communicate], could be communicated in just a letter-to-editor or brief communication (and not a full-length paper/article).

*Response: We have had extensive discussions in our research group about how to communicate the findings from this study. We agree that the lessons learned could be summarized rather briefly. However, our conclusion is that a full-length manuscript is the best option. A brief communication has its advantages but it would also mean that we would have to exclude many important aspects of the trial (e.g. the rigorous study procedures, both feasibility efficacy and process data, in-depth discussion about organizational aspects and ideas for future research and clinical policy for early interventions for trauma).*

**Reviewer #2:**

1. This is an important study that describes an early intervention for PTSD. Although the study was not completed, the results are important in this field given the paucity of studies.

*Response: We thank Reviewer #2 for pointing out the importance of these types of studies.*

2. The results and methodology are not completely in sync in terms of previous psychological trauma and psychiatric disorder. How were these assessed? Were participants with current PTSD (ie relating to a previous event) excluded from the study? If not, why not?

*Response: We agree with Reviewer #2 that this needs more clarification. As previously shown, exposure to psychological trauma is highly frequent and as much as one-third of the general population will during their life-time experience at least four potentially traumatic events [4]. As reported in the manuscript (table 1, page 12) we assessed history of traumatic events and has clarified that we used a structured assessment interview for this purpose (page 5 under Procedures, first paragraph). Previous traumatic events were not an exclusion criterion. However, a limitation of our study was that we did not assess for current psychiatric disorders and we do not know how many participants that might have fulfilled criteria for PTSD at baseline – this has been added to the limitations (page 18, first paragraph). The clinicians in the current study were very careful to assess the current index trauma (i.e. symptoms that occurred after the incident that lead to the emergency department visit) when scoring the CAPS-5. Participants who reported distress due to exposure to another previous traumatic event received an additional visit to assess symptoms related to this particular event (this extra visit occurred only in one case). Participants were also explicitly instructed to have the current index event in mind when completing the self-rated PCL-5 questionnaire. We have clarified that in the manuscript (page 6, under Measures, first paragraph).*

3. Please provide more information regarding the follow up assessments - were those who attended follow up different from those who did not?

*Response: Reviewer #2 raises an important question. Given the low number of recruited participants we did not conduct any statistical analysis. At the 2 months-follow up there was no gender difference in attendance, but at 6-months follow up we saw a small difference, 30 % (6/20) of the recruited women came back and 41% (5/12) of the recruited men. We have added this information on page 14, last paragraph.*

The authors mention sleep but this is not elaborated upon in the discussion given the literature regarding memory consolidation and sleep this is important.

*Response: We thank the reviewer for this suggestion, and we elaborate briefly on the role of sleep in early interventions for trauma in the discussion (page 16, last paragraph).*

4. The authors do not cover the general field of early intervention of PTSD. The results of the few studies that have been carried out in the first 72 hours, taken together, are not particularly encouraging. Most of the studies of early intervention have concluded that it is most effective with symptomatic survivors, not whole populations, and the few studies examining very early (ER based) interventions are not consistent. This is not reflected in the manuscript, neither in the introduction "One way to decrease the prevalence of PTSD is to intervene before the disorder develops" nor in the discussion, where the recommendation for the use of Internet based interventions or memory consolidation is made without examining the studies that do not support these results. The complexity of this issue should be discussed properly. Additionally the inclusion of participants more than 72 hours after the event raises questions about the use of memory consolidation that should be clarified.

*Response: We agree with Reviewer #2 that this issue needs to be clearer in the manuscript and we have elaborated on the current literature in the Discussion (page 17, first paragraph). We have also added to the introduction the findings from a recent study on early intervention (page 3, second paragraph).*

5. The authors state that a number of reasons led to the premature end to the study, but only mention one.

*Response: We thank Reviewer #2 for making us aware of that. The reason for the termination of the trial was one, namely organizational changes in the recruiting site that affected the number of eligible participants in a negative way. We have clarified that in the manuscript (page 4, first paragraph and in the results section).*

 While revising your submission, please upload your figure files to the Preflight Analysis and Conversion Engine (PACE) digital diagnostic tool, [https://pacev2.apexcovantage.com/](https://eur01.safelinks.protection.outlook.com/?url=https%3A%2F%2Fpacev2.apexcovantage.com%2F&data=02%7C01%7Cmaria.bragesjo%40ki.se%7C7ab645b220794d00688308d812d0d57a%7Cbff7eef1cf4b4f32be3da1dda043c05d%7C0%7C0%7C637280036169524493&sdata=VnPKTpLLQ8rYeH4vqPW2xFHfVgTjv44xwAEHnrHuXhQ%3D&reserved=0). PACE helps ensure that figures meet PLOS requirements. To use PACE, you must first register as a user. Registration is free. Then, login and navigate to the UPLOAD tab, where you will find detailed instructions on how to use the tool. If you encounter any issues or have any questions when using PACE, please email PLOS at [figures@plos.org](mailto:figures@plos.org). Please note that Supporting Information files do not need this step.

1.	Iyadurai L, Blackwell SE, Meiser-Stedman R, Watson PC, Bonsall MB, Geddes JR, et al. Preventing intrusive memories after trauma via a brief intervention involving Tetris computer game play in the emergency department: a proof-of-concept randomized controlled trial. Molecular psychiatry. 2018;23(3):674-82. Epub 2017/03/30. doi: 10.1038/mp.2017.23. PubMed PMID: 28348380; PubMed Central PMCID: PMCPMC5822451.

2.	Maples-Keller JL, Post LM, Price M, Goodnight JM, Burton MS, Yasinski CW, et al. Investigation of optimal dose of early intervention to prevent posttraumatic stress disorder: A multiarm randomized trial of one and three sessions of modified prolonged exposure. Depression and anxiety. 2020. Epub 2020/04/06. doi: 10.1002/da.23015. PubMed PMID: 32248637.

3.	Rothbaum BO, Kearns MC, Price M, Malcoun E, Davis M, Ressler KJ, et al. Early intervention may prevent the development of posttraumatic stress disorder: a randomized pilot civilian study with modified prolonged exposure. Biol Psychiatry. 2012;72(11):957-63. Epub 2012/07/07. doi: 10.1016/j.biopsych.2012.06.002. PubMed PMID: 22766415; PubMed Central PMCID: PMCPMC3467345.

4.	Benjet C, Bromet E, Karam EG, Kessler RC, McLaughlin KA, Ruscio AM, et al. The epidemiology of traumatic event exposure worldwide: results from the World Mental Health Survey Consortium. Psychol Med. 2016;46(2):327-43. Epub 2015/10/30. doi: 10.1017/s0033291715001981. PubMed PMID: 26511595; PubMed Central PMCID: PMCPMC4869975.
