# Supplementary material for: Prevention of post-traumatic stress disorder: Lessons learned from a terminated RCT of prolonged exposure
Source: PLoS One. 2021 May 24;16(5):e0251898. doi: 10.1371/journal.pone.0251898 (PMC8143412; doi:10.1371/journal.pone.0251898)
Supplement: Attachment — Submitted filename: Response to reviewer 4.docx [file pone.0251898.s007.docx]

PONE-D-20-01989

Dear Editor,

First, we want to thank you and Reviewer #4 for encouraging feedback. We have revised the manuscript accordingly. Below follows a detailed description of the changes made point by point:

**Reviewer #4**

1. The current manuscript reports data from a terminated RCT of a brief trauma focused intervention aimed at preventing the onset of posttraumatic stress disorder. The RTC was terminated due to structural changes within the hospital that led to low recruitment rates. The authors provide recommendations for improving recruitment and delivery of early interventions for PTSD in the context of issues that were encountered in the present study. The original design of the study included many strengths such as the inclusion of an active control therapy, adaptation of an existing evidenced based treatment for PTSD, and longer follow up of participants than previous studies. The authors have largely addressed the concerns of the previous reviewers.

*Response: We thank Reviewer #4 for highlighting the strengths of the original study design.*

2. The current study reports two main methodological issues: low recruitment rates (largely due to hospital infrastructure) and high attrition (not due to hospital reorganizational changes). The authors primarily provide recommendations for addressing the first issue, but not the second. It would be helpful is recommendations for each of these issues were more specifically specified.

*Response: As Reviewer #4 points out, our main issues in the study were the low recruitment rate and the high data attrition levels. We have clarified our recommendations to solves these issues in further studies in the Discussion section (eg. page 17, second paragraph). All changes have been highlighted in red.*

3. More detail could be provided about the control psychotherapy condition, such as the content that was delivered at each treatment session.

*Response: We have clarified the content of the control psychotherapy condition in the Control group section (page 11, first paragraph).*

4. With regards to recruitment and retention rates, it is unclear how these rates compare to previous studies that have used similar methods. Are the issues specific to studies recruiting individuals with trauma? Are the issues specific to the hospital at which the study took place?

*Response: Important point raised by Reviewer #4. This is indeed a common problem also seen in other trials on early interventions after trauma exposure [1-3] and we have clarified this in the Discussion (page 18, first paragraph).*

5. Regarding comment #4 or original reviewer #2, I agree that more background on early intervention for PTSD as well as internet delivery could be provided. Some of these details are already included in the Supplement research plan, but could be added to the main text. For example, the authors could note issues with previous brief interventions (e.g. Debriefing). A fuller discussion of the timing chosen for the present study and recommendations could similarly be helpful. It is not clear how treatment within 72 hours addresses the proposed theory of memory consolidation when it is stated that this occurs within 6 hours of an event, and the Rothbaum study used a different time frame (12-24 hours). The authors could discuss strategies and limitations for treating patients that may be hospitalized for acute injuries within such a short time frame. Finally, the authors could add additional research about the use of internet based treatment.

*Response: We agree with Reviewer #4 and we have added a section in the Introduction on debriefing and a short update on the current status on early interventions after trauma (page 3, second paragraph). We have also clarified the reason for the time-criterium of 72 hours used in the Rothbaum et al (2012) trial [1] (page 4, first paragraph). Our aim with our study was to replicate and extend these findings and we therefore used the same time-criterium for inclusion (page 4, second paragraph). In the Discussion, we also discuss strategies and limitations for providing hospitalized trauma victims within such a short time frame (page 18, second paragraph) and the use of internet-based treatment (page 17, second paragraph).*

6. Provide the baseline CAPS and PCL-5 scores for both groups.

*Response: Baseline symptom levels for PTSD was not assessed in this current trial due to the long recall time of CAPS and PCL-5 (CAPS-5 is to be used at the earliest one month after exposure to trauma). The only trauma-related baseline measure used in this study was the Immediate Stress Reaction Checklist (see table 1, page 14).*

6. Minor – there is an extra indent on lines 192, 312 and 373.

*Response: Adjusted accordingly.*

1.	Rothbaum BO, Kearns MC, Price M, Malcoun E, Davis M, Ressler KJ, et al. Early intervention may prevent the development of posttraumatic stress disorder: a randomized pilot civilian study with modified prolonged exposure. Biol Psychiatry. 2012;72(11):957-63. Epub 2012/07/07. doi: 10.1016/j.biopsych.2012.06.002. PubMed PMID: 22766415; PubMed Central PMCID: PMCPMC3467345.

2.	Maples-Keller JL, Post LM, Price M, Goodnight JM, Burton MS, Yasinski CW, et al. Investigation of optimal dose of early intervention to prevent posttraumatic stress disorder: A multiarm randomized trial of one and three sessions of modified prolonged exposure. Depression and anxiety. 2020. Epub 2020/04/06. doi: 10.1002/da.23015. PubMed PMID: 32248637.

3.	Iyadurai L, Blackwell SE, Meiser-Stedman R, Watson PC, Bonsall MB, Geddes JR, et al. Preventing intrusive memories after trauma via a brief intervention involving Tetris computer game play in the emergency department: a proof-of-concept randomized controlled trial. Molecular psychiatry. 2018;23(3):674-82. Epub 2017/03/30. doi: 10.1038/mp.2017.23. PubMed PMID: 28348380; PubMed Central PMCID: PMCPMC5822451.
