# Supplementary material for: Prevention of post-traumatic stress disorder: Lessons learned from a terminated RCT of prolonged exposure
Source: PLoS One. 2021 May 24;16(5):e0251898. doi: 10.1371/journal.pone.0251898 (PMC8143412; doi:10.1371/journal.pone.0251898)
Supplement: Attachment — Submitted filename: Response to reviewers.docx [file pone.0251898.s008.docx]

PONE-D-20-01989R2

Dear Editor,

We are grateful for the valuable feedback from you and Reviewer #4 and we now submit our revised manuscript for review. Below you will find a detailed description of the changes made point by point:

**Reviewer #4**

1. The present manuscript is a revised submission that provides recommendations for providing brief interventions for trauma patients to prevent the onset of PTSD. The authors have addressed the reviewers' comments.

*Response: We thank Reviewer #4 for their helpful comments and positive judgement of our revised manuscript.*

**Editor Comments**

1. We appreciate the time taken to revise this manuscript. Although there are noted improvements, it would be informative to include between-group statistics for treatment outcomes and related interpretation of any observed differences. Please see my comments on this and other issues to address, noted below:

*Response: We thank the Editor encouraging feedback. While we appreciate the editor´s feedback, we respectfully disagree on adding statistical tests. The study is not powered to detect any statistically significant changes between groups with the small sample size we ended up with. We fear that adding these analyses post hoc would be potentially misleading. Adding p-values might deflect attention from the actual size of an effect. We note that the reviewers are content with the way we have described the collected data which we feel supports our concern.*

2. Abstract: either include all section headings (background, method, results, conclusions) or no headings/paragraph form.

*Response: We have excluded the section headings in the abstract (page 2).*

3. Objective: the word “of” is omitted in “three sessions … PE” Results: should be “two-month and six-month assessments”

*Response: We have proof read the manuscript and added the word “of” on page 2 in the Abstract section and have used the word two-month and six-month assessment consequently. All changes are highlighted in red through the manuscript.*

4. Figures: In general, resolution is poor, such that the labels are unreadable in Figure 3, please change to high resolution images. Figure 2 What is described as the control condition is described as placebo here, please be consistent with naming.

*Response: We have updated the figures to a higher resolution. We have also changed the wording in figure 2 to “control group”.*

5. Introduction: “the intervention seemed to mitigate genetic predisposition to PTSD” is misleading, as psychotherapy cannot “mitigate” genetics—please rephrase to “reduce risk for PTSD development”.

*Response: We have rephrased the sentence to “reduce risk for PTSD development” (page 4, first paragraph).*

6. Method: Control group: line 229, should be “instill hope”

*Response: We have corrected to phrase to be “instill hope” (page 11, first paragraph).*

7. Table 1 should be referenced in the manuscript and a column added for relevant statistics and indication of any significant differences between intervention groups (Chi square, Fisher’s z, t statistic)

*Response: Table 1 is now referenced on page 13, second paragraph. As for the statistical tests, we refer to our response to comment no. 1.*

8. Table 2: given that 4 participants dropped out, 2 from each intervention, and the starting number is 32, shouldn’t the correct n be 13 and 15, respectively? Please also indicate the correct sample size for each group at 2 and 6 month follow up in the table, which is significantly smaller than the 32 that is listed in the top row.

*Response: We thank the editor for pointing out that the number of respondents is unclear in table 2. We have redesigned table 2 in order to clarify the number of respondents at each timepoint.*

9. Results: Even though PTSD was not assessed at baseline and sample size is small, please provide statistics both in Table 2 (in a column at the right) and in the text for all outcome measures. Although the numbers for each group are small, it is possible to examine differences in treatment outcomes, particularly with assessments that were also given at baseline (e.g., MADRS-S – repeated measures ANOVA

*Response: Please see answer to point 1. We agree that such analysis could be interesting, but fear that they would be misleading. We believe that it is possible to examine the differences by looking at the data points provided for the outcome measures and exercising judgment on the absolute differences across time points.*
